# Phenol-Soluble Modulins From *Staphylococcus aureus* Biofilms Form Complexes With DNA to Drive Autoimmunity

**DOI:** 10.3389/fcimb.2022.884065

**Published:** 2022-05-11

**Authors:** Kaitlyn Grando, Lauren K. Nicastro, Sarah A. Tursi, Jaime De Anda, Ernest Y. Lee, Gerard C. L. Wong, Çağla Tükel

**Affiliations:** ^1^ Center for Microbiology and Immunology, Lewis Katz School of Medicine, Temple University, Philadelphia, PA, United States; ^2^ Department of Bioengineering, Department of Chemistry and Biochemistry, California Nano Systems Institute, University of California, Los Angeles, Los Angeles, CA, United States

**Keywords:** PSM, Phenol Soluble Modulins, *Staphycoccus aureus*, biofilm, mesh, autoimmune disease, SLE, curli

## Abstract

The bacterial amyloid curli, produced by Enterobacteriales including *Salmonella* species and *Escherichia coli*, is implicated in the pathogenesis of several complex autoimmune diseases. Curli binds to extracellular DNA, and these complexes drive autoimmunity *via* production of anti-double-stranded DNA autoantibodies. Here, we investigated immune activation by phenol-soluble modulins (PSMs), the amyloid proteins expressed by *Staphylococcus* species. We confirmed the amyloid nature of PSMs expressed by *S. aureus* using a novel specific amyloid stain, (*E*,*E)*-1-fluoro-2,5-bis(3-hydroxycarbonyl-4-hydroxy) styrylbenzene (FSB). Direct interaction of one of the *S. aureus* PSMs, PSMα3, with oligonucleotides promotes fibrillization of PSM amyloids and complex formation with bacterial DNA. Finally, utilizing a mouse model with an implanted mesh-associated *S. aureus* biofilm, we demonstrated that exposure to *S. aureus* biofilms for six weeks caused anti-double-stranded DNA autoantibody production in a PSM-dependent manner. Taken together, these results highlight how the presence of PSM-DNA complexes in *S. aureus* biofilms can induce autoimmune responses, and suggest an explanation for how bacterial infections trigger autoimmunity.

## Introduction

Biofilms are communities of bacterial cells embedded in an extracellular matrix. Many bacterial species form biofilms, and approximately 40% of bacterial species produce amyloids within their biofilms (Larsen et al., 2007). Curli, produced by Enterobacteriaceae, including *Salmonella* species and *Escherichia coli*, are the best-characterized bacterial amyloid. Curli fibers enshroud individual bacteria as part of an extracellular matrix (ECM) to aid in biofilm surface attachment, formation of the mature biofilm architecture, and protection of the biofilm from harsh environmental conditions (Costerton et al., 1995; Reisner et al., 2003; Kikuchi et al., 2005; McCrate et al., 2013). Curli form proteinaceous fibers ranging from 4 to 12 nm in width composed of β-sheet strands oriented perpendicular to the axis of the fiber (Chapman et al., 2002; Chiti and Dobson, 2006; Schnabel, 2010), similar in structure to human amyloids like amyloid-β implicated in Alzheimer’s disease (Michelitsch and Weissman, 2000; Ghosh et al., 2021). Curli forms complexes with extracellular DNA (eDNA) in biofilms of *Salmonella enterica* serovar Typhimurium and *E. coli*. The DNA is incorporated into curli fibers, accelerating amyloid polymerization and bolstering the biofilm structure (Gallo et al., 2015).

After infections resolve, some patients develop autoimmune responses including reactive arthritis (ReA) (Carter and Hudson, 2009; Nicastro and Tükel, 2019). Moreover, individuals who have a chronic autoimmune disease like systemic lupus erythematosus (SLE) often experience disease flares when they acquire a bacterial infection (Staples et al., 1974; Shahram et al., 1993; Petri, 1998; Pachucki et al., 2020). Previous work established that the conserved quaternary structure of amyloid curli activates the heterocomplex of Toll-like receptors TLR2 and TLR1 and initiates a pro-inflammatory response (Tükel et al., 2009; Tükel et al., 2010). Curli-eDNA complexes can internalize into TLR9-containing endosomes *via* TLR2 binding (Tursi et al., 2017). Subsequent recognition of the eDNA in the curli-eDNA complex by TLR9 can lead to the production of type I interferons (IFNs) and anti-double stranded DNA (dsDNA) autoantibodies (Tursi et al., 2017). *In vitro* and *in vivo* studies suggest that amyloid curli-eDNA complexes play a role in the pathogenesis of autoimmune diseases including ReA and SLE (Gallo et al., 2015). ReA develops in about 5% of patients who suffer from gastroenteritis due to infections with enteric pathogens such as *Salmonella, Campylobacter*, *Shigella*, or *Yersinia* (Carter and Hudson, 2009). Studies using *S.* Typhimurium as a model organism revealed that the joint inflammation and autoimmune sequalae observed following infection are driven by curli (Miller et al., 2020). Furthermore, it was recently demonstrated that exposure to curli is associated with disease flares in patients with SLE (Pachucki et al., 2020). At present, the mechanistic links between enteric infections and autoimmune outcomes are not well understood.

Persistent *Staphylococcus aureus* infections are often associated with the formation of a biofilm and are of particular interest as they are recalcitrant to immune responses and unresponsive to antibiotic therapies (Boles and Horswill, 2008). Persistent infections involving *S. aureus* biofilms can lead to osteomyelitis and endocarditis (Fernández Guerrero et al., 2009; Olson and Horswill, 2013), and *S. aureus* biofilm growth often occurs on medical implants (Kathju et al., 2015; Langbach et al., 2016). Studies have shown that *S. aureus* carriage is associated with certain autoimmune diseases such as granulamatosis with polyangiitis, psoriasis, and SLE (Conti et al., 2016; Totté et al., 2016; Salmela et al., 2017; Ceccarelli et al., 2019). Further, superantigen expression by *S. aureus* is critical for the activation of autoreactive T cells in mice (Tuffs et al., 2018).

*S. aureus*, a Gram-positive pathogen, produces amyloids known as phenol-soluble modulins (PSMs) that also form complexes with DNA (Schwartz et al., 2012; Schwartz et al., 2016). PSMs are essential for proper development and stabilization of biofilms formed by *S. aureus* but also serve a role in biofilm detachment and dispersal leading to systemic dissemination of the pathogen through mechanisms that are not well-understood (Wang et al., 2007; Periasamy et al., 2012; Schwartz et al., 2012; Zaman and Andreasen, 2020). PSMs are amphipathic alpha-helical proteins that range in size from 20 to 45 amino acids in length. *S. aureus* expresses nine types of PSMs that are classified further into α and β groups (α1 to 4 and β1 and 2) and δ-toxin (Wang et al., 2007; Peschel and Otto, 2013). Recently, research has indicated that eDNA forms complexes with PSMs and promotes PSM fibrillization (Schwartz et al., 2016). PSMα3 is of particular interest as the recently solved microcrystallographic structure of PSMα3 indicated that it forms cross-α amyloid fibrils that have structures similar to those of amyloid cross-β sheet structures, as seen in curli fibers (Tayeb-Fligelman et al., 2020). High levels of fluorescence are observed when either cross-α amyloid fibrils or cross-β sheet structures bind to Thioflavin T (Tayeb-Fligelman et al., 2017). Similar to curli and other human amyloids, PSM fibrils are agonists of TLR2 (Hajjar et al., 2001; Cheng et al., 2008; He et al., 2009; Tükel et al., 2009; Chen et al., 2014). Additionally, PSMs activate the human formyl peptide receptor 2 (FPR2/ALX), which has previously been implicated in endogenous inflammatory processes (Kretschmer et al., 2010; Rautenberg et al., 2011; Kretschmer et al., 2012). Of the PSMs, PSMα3 has the highest propensity for membrane permeation and DNA binding (Laabei et al., 2014; Schwartz et al., 2016; Towle et al., 2016), which can both lead to inflammation.

Here, we examined whether chronic infection with *S. aureus* biofilms would drive autoimmune responses *via* the production of PSM-eDNA complexes, similar to autoimmune responses triggered by curli-eDNA complexes. We found that intraperitoneal injection of synthetic PSMα3 fibrillized with CpG DNA into mice elicits a robust anti-dsDNA autoantibody response. To assess whether similar outcomes can be precipitated using an infection model, we showed that implantation of a mesh-associated *S. aureus* biofilm into a mouse directly leads to induction of anti-dsDNA autoantibodies, and importantly, does so in a TLR2 and TLR9 dependent manner. In contrast, intraperitoneal infection with *S. aureus* wildtype SH1000 or Δ*psm* mutant does not induce autoantibody production. These results indicate that both PSMα3 and DNA are involved in the induction of autoantibodies. Moreover, the mechanisms of entry and immune activation implicate both TLR2 and TLR9, which suggest that PSMα3-DNA complexes and curli-DNA complexes behavior similarly in immune activation. These results using either *S. aureus* biofilm infection or synthetic PSMs illustrate how bacterial biofilms can lead to infection-associated autoimmunity.

## Results

### *S. aureus* Biofilms Contain PSMs

PSMs are secreted by *Staphylococcus* in high amounts in a quorum-sensing-controlled fashion (Kretschmer et al., 2012). The role of PSMs in the structuring and stabilizing of biofilms due to their amyloid conformation had been controversial, but recently it was reported that PSMs are crucial for formation of *S. epidermidis* biofilms on indwelling devices (Le et al., 2019). *S. aureus* PSMs form fibrillar structures *in vitro* that resemble curli fibrils, and, like curli, PSMs bind DNA (Schwartz et al., 2016). To characterize PSM-containing biofilms, we first examined *S. aureus* SH1000 and its isogenic *psm* mutant that lacks all six PSMα- and β-encoding genes. We utilized a Congo red derivative (*E*,*E)*-1-fluoro-2,5-bis(3-hydroxycarbonyl-4-hydroxy) styrylbenzene (FSB) that specifically bind amyloids to study the biofilms formed by these strains. FSB was synthesized for specific detection of amyloid-β in Alzheimer’s disease brain tissue with little background staining (Sato et al., 2004). When tested on *E. coli* UTI89, a uropathogenic clinical strain whose biofilm extracellular matrix is composed primarily of curli and cellulose, unlike Congo red, FSB stained curli but not the carbohydrate cellulose, supporting that FSB specifically stains amyloidogenic proteins (Reichhardt and Cegelski, 2018).

**Figure 1:**
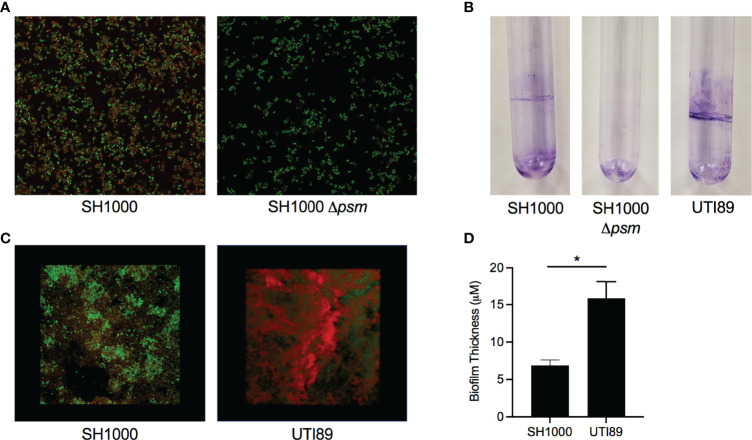
*Staphylococcus aureus* biofilms contain amyloid that can be detected by an amyloid specific stain. **(A)** Confocal Laser Scanning Microscopy (CLSM) images of *in vitro* biofilms of *S. aureus* SH1000 (lab strain) and SH1000 phenol soluble modulin mutant (Δ*psm*) were stained with syto9 (green) and FSB (red) to determine the expression of PSM amyloids. **(B)** Crystal Violet staining of pellicle biofilms grown in glass tubes of SH1000, SH1000 Δ*psm*, and UTI89 (*E. coli* clinical isolate). **(C)** CLSM surface images of *in vitro* biofilms of *S. aureus* SH1000 and *E coli* UTI89 were stained with syto9 (green) and FSB (red). **(D)** Overall biofilm thickness of SH1000 and UTI89 biofilms as determined by Leica TCS imaging software. Mean and SEM graphed; significance was calculated using Unpaired t test (*, P < 0.05).

*S. aureus* SH1000 biofilms were grown in peptone-NaCl-glucose (PNG) media while UTI89 biofilms were grown in LB no salt as described previously, to compare amyloid staining between PSMs and curli (Schwartz et al., 2012; Tursi et al., 2020). *S. aureus* SH1000 biofilms were stained with the amyloid-specific FSB and with the nucleic-acid-specific Syto9 dye and examined with confocal microscopy. We found that FSB stained the *S. aureus* SH1000 biofilm (**Figure 1A**, left), while there was minimal visible FSB staining of the *Δpsm* mutant (**Figure 1A**, right). This small amount of staining may be due to the presence of other amyloidogenic proteins in the *S. aureus* extracellular matrix, such as additional PSMs, which were not deleted in our *Δpsm* mutant and has been shown to form amyloid fibrils *in vitro* (Zhou et al., 2021). Next *S. aureus* SH1000, its isogenic *psm* mutant, and UTI89, which expresses curli, were grown in sterile glass tubes and pellicles were stained by crystal violet. SH1000 and UTI89 formed visible pellicle biofilms, while SH1000 *Δpsm* did not (**Figure 1B**). Although SH1000 biofilms showed FSB staining throughout the biofilm (**Figure 1C**), when 3D images of SH1000 were compared to images of UTI89 biofilms, SH1000 biofilms were less compact (**Figure 1C**) and significantly thinner (**Figure 1D**), highlighting that *S. aureus* PSMs are an integral part of the biofilm structure. However, these results also suggested that although PSMs are expressed in the PNG limited media, it might not be the optimal *in vitro* conditions for PSM expression or biofilm formation.

**Figure 2:**
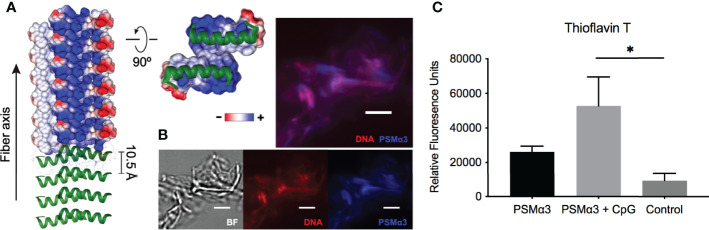
DNA promotes PSM fibrillation *via* direct association. **(A)** Crystal structure of PSMα3 fiber (PDB: 5I55) with electric potentials of charge residues projected on surface model. The electric potentials were calculated using Chimera software. **(B)** Fluorescence staining of PSMα3-DNA complexes using nucleic acid dye BOBO-3 iodine (red) and Thioflavin T, which only binds to fibrillar amyloid structures. Scale bar: 3μm.**(C)** Fibrillization of synthetic PSMα3 with or without CpG DNA was monitored by Thioflavin T fluorescence and reported as relative fluorescent units (RFU). Mean and SEM graphed; significance was calculated using One-way ANOVA with Tukey’s multiple comparisons test (*, P < 0.05).

Like curli-eDNA complexes, *S. aureus* PSMs have also been shown to bind DNA (Schwartz et al., 2016). However, unlike the net negatively charged β-sheet subunits of curli fibers, the α-helical PSMs (PSMα1-4) are amphiphiles with net positive charge (Peschel and Otto, 2013). For instance, the recently published crystal structure of the cross-α fibers formed by PSMα3 (Tayeb-Fligelman et al., 2020) indicate the positions of cationic charges along the fibrillation axis on the hydrophilic surface of the PSM fiber (**Figure 2A**). The existence of high local cationic charge densities suggest potential binding sites for dsDNA (Wong, 2006; Wong and Pollack, 2010). To visualize direct interaction of the two oppositely charged polyelectrolytes, we incubated synthetic PSMα3 with bacterial genomic DNA for 24 hours for fibrillation. At the end of the incubation period, the fluorescent dye Thioflavin T, which only binds to fibrillar amyloid structures, and the nucleic acid dye BOBO-3 iodide, were added to the solution for fluorescent imaging of the microscopic PSM-DNA complexes (**Figure 2B**). We observed fibrillar particles with overlapping fluorescent signal of DNA and PSM amyloid dyes that confirm their direct molecular interaction. To examine whether DNA can promote the formation of fibrillar structures (Towle et al., 2016), we incubated synthetic PSMα3 with or without CpG DNA, unmethylated oligonucleotides containing CpG motifs for TLR9 binding to simulate the unmethylated CpG-rich bacterial DNA (Bird, 1987), and monitored fibrillization by incubating with Thioflavin T. We found that PSMα3 polymerizes into a fibrillar amyloid structure to a greater extent in the presence of DNA compared to PSMα3 alone (**Figure 2C**). These results highlight the similarities of PSMs and curli: both are naturally produced in biofilm formation; both can be stained by amyloid-specific dyes; and both involve DNA in fibrilization processes.

### Systemic Presence of Synthetic PSMα3-DNA Complexes Elicits an Autoantibody Response

**Figure 3:**
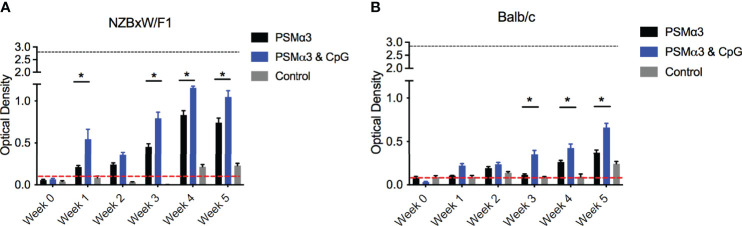
Intraperitoneal injection of synthetic PSM⍺3 fibrillized with CpG DNA elicits an autoantibody response. **(A)** NZBxW/F1 or **(B)** Balb/c mice were injected bi-weekly with PSMα3 fibrillzed alone (black bar) or fibrillzed in the presence of CpG DNA (blue bar) or control mice were injected with sterile PBS (gray bar). Mice were tail bled weekly and the production of anti-dsDNA autoantibodies were detected (Optical density 650-405nm). Black dotted line represents the maximum anti-dsDNA autoantibody production of positive control sera and the red dotted line represent autoantibody detection in naïve serum. Mean and SEM graphed; significance was calculated using a 2-way ANOVA and Tukey’s multiple comparisons tests (*, P < 0.05).

Complexes of curli and eDNA induce anti-dsDNA and anti-chromatin autoantibody production in mouse models (Gallo et al., 2015). We therefore tested whether PSMα3 peptide, fibrillized with and without CpG DNA, would induce autoantibody responses in wild-type Balb/C or in autoimmune disease-prone NZBXW/F1 mice. Mice were injected intraperitoneally twice per week with PSMα3 alone, PSMα3 fibrillized with CpG DNA, or PBS as a negative control. Then anti-dsDNA autoantibodies were monitored in the serum compared to the highly autoimmune MRL/lpr mouse sera as a positive control. NZBXW/F1 mice treated with either PSMα3 or PSMα3-DNA showed increased production of autoantibodies compared to PBS-treated mice (**Figure 3A**). PSMα3-treated and PSMα3-DNA-treated mice produced autoantibodies as early as 1 week after the first injection (**Figure 3A**). Between weeks 4 and 5, autoantibody production appeared to plateau. The level of autoantibody production induced by PSMα3 fibrillized with CpG DNA did not reach the level induced by MRL/lpr sera but was significantly higher than the amount produced by mice injected with PSMα3 alone. In Balb/C mice, we again observed significantly more autoantibody production upon treatment with the PSMα3-DNA complex than the mice treated with PSMα3 alone (**Figure 3B**). Although the autoantibody response in Balb/C mice was lower than in the autoimmune disease-prone mice, these data indicate that the PSMα3-DNA complex is more immunogenic than PSMα3 alone and has the capacity to elicit an autoantibody response both in wild-type mice and mice predisposed to developing autoimmunity.

### *S. aureus* Colonized Mesh Implantation Induces Autoantibody Production

**Figure 4:**
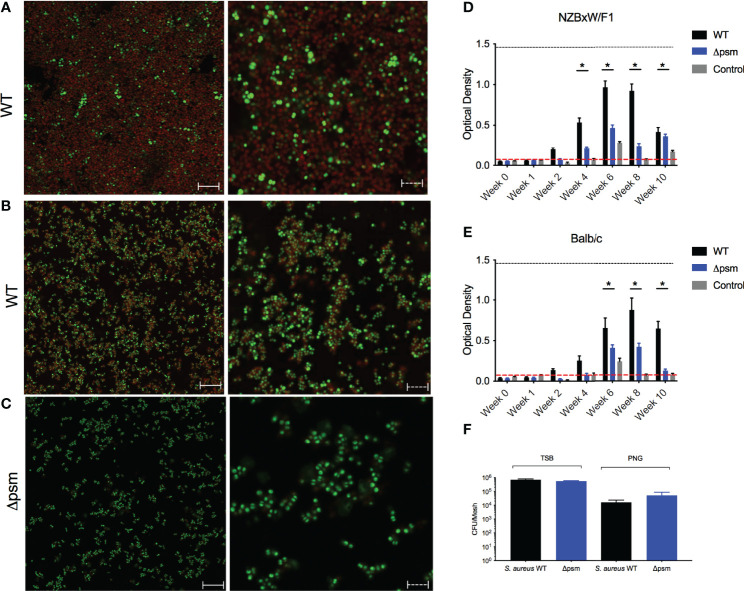
*S. aureus-*colonized mesh implantation induces autoantibody production. To determine the expression of PSM amyloids, Confocal Laser Scanning Microscopy (CLSM) images of *in vitro* biofilms of *S. aureus* ***(*A*)*** SH1000 (WT) and **(C)** Δ*psm* SH1000 stained with syto9 (green) and Congo red (red); or **(B)** SH1000 (WT) stained with syto9 (green) and amyloid-specific dye 1-Fluoro-2,5-bis[(E)-3-carboxy-4-hydroxystyryl]benzene (FSB). Biofilms visualized at 100x, solid scale bar represents 50µM and dashed scale bar represents 5µM. *S. aureus* WT (black bars) or Δ*psm* (blue bars) biofilm-colonized mesh or control mesh was implanted subcutaneously into the back flanks of **(D)** NZBxW/F1 or **(E)** Balb/c mice. Blood was collected *via* tail bleeding every week and sampled for production of dsDNA autoantibodies (optical density 650-405nm). Black dotted line represents the maximum autoantibody production of positive control sera and the red dotted line represents autoantibody detection in naïve serum. Mean and SEM graphed; significance was calculated using a 2-way ANOVA and Tukey’s multiple comparisons tests (*, P < 0.05). **(F)** Biofilms of *S. aureus* SH1000 wildtype (WT) (black bar) or Δ*psm* mutant (blue bars) were grown on mesh in either tryptic soy broth or peptone-based media (PNG) for 48 hours at 37°C. Biofilms were sonicated and the recovered bacteria were enumerated as colony forming units. Mean and SEM graphed; significance was calculated using a 1-way ANOVA and multiple comparisons tests. No statistical significance was determined.

We next investigated the effect of biofilm-associated *S. aureus* infection of mice on autoantibody production. As *Staphylococcus* species can colonize surgical mesh and cause infections following surgeries in humans, we grew either *S. aureus* SH1000 or the Δ*psm* mutant under biofilm-inducing conditions on sterile surgical mesh (1x1 mm) for 48 hours at 37°C. Biofilms were stained either with amyloid specific dyes Congo red (**Figure 4A**) or FSB (**Figure 4B**). Amyloid staining was observed both with Congo red and FSB, but the *S. aureus* psm mutant did not show any staining, as expected (**Figure 4C**). To confirm that the mesh could be colonized with equal numbers of wild-type and mutant SH1000, the bacteria were recovered by sonication and enumerated on tryptic soy agar; bacterial numbers confirmed that *S. aureus* SH1000 and the Δ*psm* mutant can colonize the mesh and form biofilms that contain equal numbers of bacteria (**Figure 4F**), even though the robustness of biofilm is different due to their difference in ECM (**Figures 4A-C**).

Biofilm-associated mesh were then inserted into the back flanks of anesthetized mice similar to a catheter insertion surgery that we developed recently (Tursi et al., 2020). After implantation, sera samples were taken weekly or biweekly for 10 weeks, and anti-dsDNA autoantibodies and bacterial CFU were quantified. In NZBxW/F1 mice, implanted with the mesh carrying *S. aureus* SH1000 biofilm, autoantibodies were detected at 4 weeks post-implantation (**Figure 4D**). Although anti-dsDNA antibodies were detected in NZBxW/F1 mice that had been implanted with mesh colonized with Δ*psm* (**Figure 4D**), the levels of antibodies were significantly lower, indicating the importance of the PSM component of the biofilm in production of anti-dsDNA autoantibodies. In the Balb/C mice, the response to the wild-type biofilm implant was slightly delayed relative to that in autoimmune disease-prone mice with only marginal production at 4 weeks, and the extent of anti-dsDNA antibody production was lower in mice with Δ*psm*-colonized mesh implants (**Figure 4E**). In both NZBxW/F1 and Balb/C mice, anti-dsDNA autoantibodies declined after week 8. Unlike humans, *S. aureus* cannot establish long-term infections in mice and are cleared. Analyses of blood taken from these mice showed that active infection was undetectable after 2 weeks (**Figure S1**). The results showing that anti-dsDNA autoantibodies persist in circulation, even after bacteria can no longer be detected, suggest that the immune system detects the PSM-DNA complexes on *S. aureus* biofilms and respond to the DNA.

### Autoantibody Production Induced by *S. aureus*-Colonized Mesh Implants Are Dependent on TLRs

**Figure 5:**
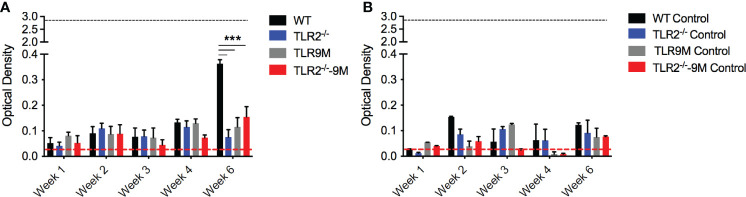
*S. aureus-*colonized mesh implantation induces autoantibody production dependent on TLRs. **(A)** *S. aureus* WT colonized mesh or **(B)** control mesh was implanted subcutaneously into the back flanks of C57BL/6 wildtype (WT) (black bars), TLR2^-/-^ (blue bars), TLR9 mutant (TLR9M) (gray bars), and TLR2^-/-^ - TLR9M (TLR2^-/–^9M) (red bars) mice. Blood was collected *via* tail bleeding every week and sampled for production of anti-dsDNA autoantibodies (optical density 650-405nm). Black dotted line represents the maximum autoantibody production of positive control sera and the red dotted line represent autoantibody detection in naïve serum. Mean and SEM graphed; significance was calculated using a 2-way ANOVA and Tukey’s multiple comparisons tests (***, P < 0.001).

PSM-DNA complexes induced a TLR-dependent immune response *in vitro*. To confirm that the autoantibody production in response to biofilm-associated mesh depends on TLRs, we performed mesh implantation experiments in wild-type C57BL/6 mice and in mice lacking TLR2 or TLR9 or both. SH1000-colonized mesh and control sterile mesh were implanted subcutaneously in the back flanks of the mice. At week 6 after implantation with SH1000-colonized mesh, we observed significantly higher levels of autoantibodies in the wild-type mice compared to the TLR-deficient mice (**Figure 5A**). Little or no autoantibody production was detected in mice implanted with sterile mesh, and no significant differences were detected between wild-type and mutant mice at any time point (**Figure 5B**). Thus, the autoimmune responses are dependent on biofilm colonization of the mesh and are TLR-dependent.

### Intraperitoneal Injection of *S. aureus* Does Not Induce Autoantibody Production

**Figure 6:**
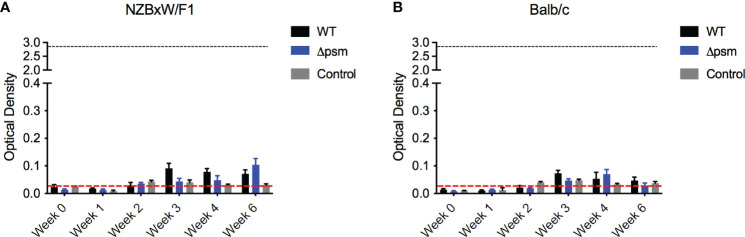
Intraperitoneal infection with *S. aureus* wildtype SH1000 or Δ*psm* mutant does not induce autoantibody production. **(A)** NZBxW/F1 or **(B)** Balb/c mice were injected intraperitoneally with 10^7^ *S. aureus* WT (black bars) or *psm* mutant (Δ*psm*) (blue bars) or PBS-injected control mice (gray bars). Serum was collected weekly to measure the levels of anti-dsDNA autoantibody production (optical density 650-405nm). Black dotted line represents the maximum autoantibody production of positive control sera and the red dotted line represent autoantibody detection in naïve serum. Mean and SEM graphed; significance was calculated using a 2-way ANOVA and Tukey’s multiple comparisons tests. No statistical significance was determined.

As acute staphylococcal infections are not associated with autoimmunity, we hypothesized that the longer exposure to PSM-containing *S. aureus* biofilm rather than the acute bacterial infection would drive the autoantibody response in mice. To test this, we injected Balb/C and NZBxW/F1 mice intraperitoneally with 10^7^ colony-forming units (CFU) of *S. aureus* SH1000, the isogenic Δ*psm* mutant, or with PBS as a negative control, and tracked autoantibody production by analysis of mouse sera. Blood was sampled for evaluation of systemic bacteria by plating on tryptic soy agar plates. Overall bacterial numbers were low and below the detection limit in both mouse strains, indicating *S. aureus* clearance almost immediately after infection. In both Balb/C and NZBxW/F1 mice, the levels of anti-dsDNA autoantibodies were low upon injection of either strain of *S. aureus* and comparable to PBS injections (**Figures 6A, B**). Together, these data show that acute exposure to PSMs in not sufficient to generate an autoimmune response. Our previous data above showed that chronic exposure to synthetic PSMα3 in complex with DNA (**Figure 3**), or to PSM-containing *S. aureus* biofilm (**Figure 4**), induces autoantibody production, suggesting that chronic biofilm infections with *S. aureus* rather than acute bacterial infections may drive the autoantibody response in humans.

## Discussion

In this study, we demonstrated striking similarities between the PSMs of *S. aureus* and the well-characterized bacterial amyloid curli. Curli fibers are the major proteinaceous component of several enteric bacterial biofilms, including *E. coli* and *Salmonella*, and play a pivotal role in the three-dimensional structure of the ECM (Olsén et al., 1989; Costerton et al., 1995; Larsen et al., 2007; Hung et al., 2013; McCrate et al., 2013; Tursi and Tükel, 2018). Curli provides strength to the ECM mainly by its amyloid properties, such as resistance to enzymatic degradation and physical stress. Amyloid proteins are known to bind Congo red dye but recent studies have shown that other carbohydrates, like cellulose in the ECM, can also bind Congo red (Reichhardt and Cegelski, 2018). Therefore, we utilized the Congo red derivative, FSB stain, which specifically binds the amyloid component of the biofilms allowing studies on amyloids to be more specific (Sato et al., 2004; Reichhardt and Cegelski, 2018). Previous studies showed that PSMs produced by *S. aureus* form curli-like amyloid structures (Schwartz et al., 2012; Schwartz et al., 2016). However, the studies on PSMs have been controversial, as these proteins play multiple functions during the lifestyle of *Staphylococcus* species and within their biofilms. In this study, we determined that when, wild-type *S. aureus* SH1000 biofilms are grown in PNG media, unlike traditional biofilms formed by *S. aureus*, SH1000 formed visible pellicles at the air-liquid interphase, while the Δ*psm* mutant did not (**Figure 1B**). PSMs also stained with the amyloid-specific FSB stain, similar to the *E. coli* UTI89 strain that expresses curli (**Figures 1A, C**). However, the biofilms formed by *S. aureus* SH1000 were not as robust as *E. coli* UTI89, indicating differences in biofilm architecture between PSM-containing and curli-containing biofilms.

Both curli and PSMs bind DNA in the ECM (Gallo et al., 2015; Schwartz et al., 2016; Towle et al., 2016; Tayeb-Fligelman et al., 2017). The periodicity of cationic helical subunits along the PSMα3 cross-α amyloid fibers (Tayeb-Fligelman et al., 2020) present a cationic scaffold for interaction with the highly negatively charged DNA (**Figure 2A**), cognate to recent characterization of protofibrils formed by cationic amphiphilic antimicrobial peptides when complexed with DNA (Lee et al., 2020). Previous studies have determined that bacterial DNA promotes polymerization of curli. Consistent with these findings, synthetic PSMα3 formed increased fibrillar structures in the presence of DNA (**Figures 2B, C**). These results support the hypothesis that PSMs act as amyloids, with a critical role in biofilm formation and the extracellular matrix of *S. aureus* biofilms (Schwartz et al., 2012; Schwartz et al., 2016).

There is evidence that infectious agents can trigger gut or systemic autoimmune diseases as well as autoimmune disease flares. Although controversial, bacterial biofilms on implanted devices, such as surgical mesh, have been implicated in coercion of the immune system into breaking tolerance and inducing autoimmune diseases (Chughtai et al., 2017a; Chughtai et al., 2017b; Clancy et al., 2019; Strietzel et al., 2019). Many bacteria that colonize implanted devices have the capacity to produce amyloids (Nicastro and Tükel, 2019). Recent work showed the presence of multiple-species biofilms containing staphylococcal species on implanted mesh (Kathju et al., 2015; Langbach et al., 2016; Le et al., 2019). *In vivo* intraperitoneal injection of PSMα3, with and without DNA, or subcutaneous implantation of SH1000 *S. aureus* biofilms grown on mesh, and not the Δ*psm* mutant biofilms, induced a significant anti-dsDNA autoantibody response, especially in autoimmune-prone mice (**Figures 3**, **4**). This response was TLR-dependent, with WT mice producing a significant autoantibody response while there was no significant response in TLR2- and TLR9-deficient mice (**Figure 5**). Though we did not test TLR-dependence in autoimmune-prone mice, we would expect similarly accelerated autoantibody response to mesh implantation, which would be ameliorated in TLR-deficient mice, but this requires further testing to determine if TLRs are the only receptors responsible when other risk factors for autoimmunity are present. Meanwhile intraperitoneal injection of planktonic *S. aureus*, regardless of PSMs, did not provoke an autoantibody response (**Figure 6**), emphasizing that only chronic biofilm infections containing PSMs induce such a response.

For the first time, we have observed that even without DNA, fibrillar PSMα3 alone was able to induce low levels of anti-dsDNA autoantibodies. As it was previously reported that PSMα3 is cytotoxic to immune cells (Wang et al., 2007; Cheung et al., 2014; Laabei et al., 2014; Tayeb-Fligelman et al., 2017), the autoimmune response generated following systemic injections of mice with only PSMα3 could be due to the formation of complexes of PSMα3 with eukaryotic DNA released from the lysed cells. It is also plausible that PSMs form cytotoxic intermediate fibrils, like those of curli which were recently reported to occur during the early stages of biofilm formation (Nicastro et al., 2019). At the same time, it is unlikely that the presence of CpG DNA alone induced the autoantibody response: previous studies have shown that BALB/C mice or C57BL/6-*lpr/lpr* mice immunized with CpG DNA alone did not produce anti-dsDNA autoantibodies, but instead CpG DNA worked as an adjuvant to enhance the immune response by stimulating TLR9 (Tran et al., 2003; Lartigue et al., 2006).

Overall, the anti-dsDNA autoantibody levels in these experiments were relatively low compared to what was observed in response to curli-DNA complexes (Gallo et al., 2015; Tursi et al., 2017; Miller et al., 2020). We noted the rapid clearing of *S. aureus* in our model (**Supplementary Figure 1**), which could lead to the observed decrease in anti-dsDNA autoantibodies. It is our expectation that chronic exposure to *Staphylococcus* species that can form robust biofilms, with high loads of PSMs and DNA in their ECM, could break tolerance and generate stronger and sustained autoimmune response. Together, these results corroborate studies suggesting a pro-inflammatory role of PSMs (Wang et al., 2007; Syed et al., 2015; Nakagawa et al., 2017), which appears to be TLR-dependent (Hajjar et al., 2001; Hanzelmann et al., 2016; Schlatterer et al., 2018), and similar to curli, associated with the generation of a type I IFN and autoimmune response (Gallo et al., 2015; Tursi et al., 2017), though the exact mechanism remains to be determined.

Despite the similarities observed between curli and PSMα3 in the *in vitro* and *in vivo* experiments performed in this study, it is important to note that literature remains controversial on the topic of whether the amyloidogenic properties of PSMs are indeed critical and play a multi-functional role in *S. aureus* biofilms. Several studies contest that rather than forming the structure of the extracellular matrix, like curli does for *E. coli*, PSMs are responsible for dispersal of the biofilm and dissemination to other areas (Kong et al., 2006; Otto, 2008; Dastgheyb et al., 2015). A role for PSM amyloids was also implicated in creating the characteristic channels in mature *S. aureus* biofilms that allow nutrient distribution to the deeper regions of the biofilm, and in regulating the dynamic waves of detachment and systemic spread from mature biofilms by acting as surfactants to break up the extracellular matrix (Periasamy et al., 2012; Le et al., 2014). One such study acknowledges the amyloidogenic properties of PSMs *in vitro*, but noted that there appears to be no correlation between the amyloidogenicity of PSMs and their role in biofilm extracellular matrix structure nor inflammatory response (Zheng et al., 2018). We find that this is not inconsistent with our expectations: given that DNA and PSMα3 self-assemble into an electrostatic complex, their structure and stability can be influenced by solution conditions, as will their downstream inflammatory capacity. This contention is echoed in studies by both groups (Schwartz et al., 2012; Schwartz et al., 2016; Zheng et al., 2018), where formation of PSM amyloids were highly condition-specific. What’s more, it is already known that PSMs are significantly regulated by quorum-sensing (Kong et al., 2006; Boles and Horswill, 2008; Le et al., 2014), so it is possible that *in vivo* in different tissues or niches, PSMs serve multiple purposes, which may be regulated by both their expression level and structure as soluble peptides that act as surfactants or as amyloid aggregates which contribute to biofilm structure.

In summary, we have demonstrated a significant correlation between PSMs in *S. aureus* biofilms and anti-dsDNA autoantibody production, a marker of autoimmunity, in mice. *Staphylococcus epidermidis* is the most common implant-associated infection; this strain is a skin commensal, but becomes an opportunistic pathogen when introduced systemically *via* indwelling devices (Otto, 2009). PSMs have also been shown to play major roles in *S. epidermidis*, similar to that seen in *S. aureus*, including pro-inflammatory functions and biofilm dispersal and structure (Mehlin et al., 1999; Wang et al., 2011). One study suggests that PSMs in *S. epidermidis* do not form amyloid structures like those in *S. aureus* but remain important for overall biofilm maturation and architecture (Le et al., 2019). Future experiments are needed to determine if there are stages during *in vivo* biofilm formation with increased amyloid PSM-DNA complexes, that can subsequently induce autoantibody responses, to resolve the role of PSM amyloidogenicity in *Staphylococcus* biofilm-associated inflammation and autoimmune disease. Alternatively, PSMs dislodging from the biofilm could be sufficient to stimulate the immune cells to create an autoinflammatory environment. Further epidemiological studies on the association between different *Staphylococcus* strains, their PSM levels and structures, and autoimmune sequelae, are needed to fully elucidate the contribution of PSMs and their mechanism of pathogenicity. Should we find that PSMs function similar to curli as critical components of *S. aureus* and/or *S. epidermidis* biofilms, therapies targeting amyloids may be pursued in order to treat these notoriously antibiotic-resistant infections.

## Materials and Methods

### Bacterial Strains and Culture Conditions

The *S. aureus* SH1000 and the Δ*psm* mutant strain were described previously (Schwartz et al., 2016). Uropathogenic *E. coli* UTI89 (isolated from a patient with an acute bladder infection) was kindly provided by Dr. Scott Hultgren from Washington University in St. Louis. Overnight cultures of SH1000 and the Δ*psm* mutant were grown in tryptic soy broth at 37°C with shaking at 200 rpm; *S.* Typhimurium IR715 *msbB* mutant was previously described (Raffatellu et al., 2005) and was grown in LB broth supplemented with 100 μg/mL kanamycin at 37°C with shaking at 200 rpm; and *E. coli* UTI89 was grown in LB broth at 37°C with shaking at 200 rpm.

### Biofilm Growth Analysis by Confocal Microscopy

Biofilms of *S. aureus* SH1000, the isogenic Δ*psm* mutant, or *E. coli* UTI89 were grown on sterile glass circular coverslips or on medical mesh by diluting overnight cultures 1:100 in PNG media for *S. aureus* strains, or LB low salt media for UTI89, for 48 hours at 37°C (Schwartz et al., 2012; Tursi et al., 2020). To visualize PSM in biofilms, coverslips were washed three times with sterile PBS and stained with 3 µg/mL Syto9 (ThermoFisher, S34854) for 10 minutes in the dark. Biofilms were then gently washed three times with sterile PBS and stained with 12.5 µM FSB (Millipore, 07602) or 10 ug/m Congo red (Sigma Aldrich, HT60-1KT) for an additional 10 minutes. Coverslips were placed upside down in 8-well Multi-test Slides (MP Biomedicals, 096040805E) with 3 μl Vectashield (Vector Labs, H-1000) between the coverslip and slide to prevent photo-bleaching. Syto9 was visualized with excitation at 483 nm and emission of 503 nm, FSB was visualized with excitation at 390 nm and emission of 511 nm, and Congo red was visualized with excitation at 561 nm and an emission of 650-750nm using a Leica TCS confocal imaging system at 63x magnification. Biofilm thickness was measured on Leica TCS imaging software.

### Crystal Violet Staining of Biofilms

Pellicle biofilms were grown and stained with crystal violet as previously described (Schwartz et al., 2012). Briefly, overnight cultures of *S. aureus* SH1000, SH1000 Δ*psm* mutant, and *E. coli* UTI89 were diluted 1:100 into 3 mL of PNG media for *S. aureus* strains or LB low salt media for UTI89 and biofilms were grown in sterile glass tubes for 48 hours at 37°C with shaking at 200 rpm. Pellicle biofilms formed at the air-liquid interface and were washed with 10 mL ddH_2_O with or without 1% SDS, vortexed briefly, and then stained with 0.1% crystal violet. Tubes were gently washed with ddH_2_O and then photographed.

### Isolation of Curli

Biofilms of *S.* Typhimurium were grown by diluting overnight cultures 1:100 in YESCA broth supplemented with 4% DMSO to enhance curli formation as previously described (Lim et al., 2012). Cultures were grown at 26°C for 72 hours with shaking at 200 rpm. Curli was isolated from biofilms as previously described (Collinson et al., 1999).

### Calculation of Electrostatic Potentials on PSMα3 Fibrils

The cross-α crystal structure of PSM⍺3 amyloid fibril was downloaded from RCSB Protein Data Bank (PDB: 5I55). The electrostatic potential surface values were calculated using UCSF Chimera software (Pettersen et al., 2004). The values are calculated according to Coulomb’s law with dielectric constant of 80 and a distance-dependent dielectric.

### Fluorescence Visualization of PSM-DNA Complexes

Synthetic PSMα3 peptide (MEFVAKLFKFFKDLLGKFLGNN) was purchased from LifeTein. PSMα3 (10 mg/ml) was fibrillized in the presence of *Escherichia coli* genomic DNA (ThermoScientific, J14380.MA) at a charge ratio of 2:1 (5 mg/ml). The solution was incubated at room temperature overnight with shaking. After the incubation period, Thioflavin T (Sigma, T3516) and BOBO-3 Iodine (ThermoScientific, B3586) dyes were added to the solution for fiber amyloid and DNA staining, at final concentration of 10 µM and 6 µM respectively. The complexes were imaged using an Andor Neo sCMOS camera with Andor IQ software on an Olympus IX83 microscope equipped with a 100x oil objective and Zero Drift Correction 2 continuous autofocus system (pixel resolution of 0.065 μm/pixel). BOBO-3 Iodine DNA staining was visualized with Lambda LS (Sutter Instrument) xenon arc lamp and a red fluorescent protein (RFP) filter, excitation at 553 nm and emission at 574 nm, with an exposure of 500ms; Thioflavin T PSMα3 amyloid stain was visualized using a cyan fluorescent protein (CFP) filter, excitation at 433 nm and emission at 475 nm, with an exposure of 250ms.

### Synthetic PSMα3 Fibrillization

Synthetic PSMα3 peptide (MEFVAKLFKFFKDLLGKFLGNN) was purchased from Biosynthesis. PSMα3 (2.5 µg/ml) was fibrillized in the presence or absence of CpG DNA (*In vivo*gen, ODN 1826) at a charge ratio of 20. Fibrillization was monitored in the presence of 10 µM Thioflavin T (Sigma, T3516) for 10 minutes protected from light. The relative fluorescent units (RFU) were detected (excitation 440 nm/emission 490 nm) using a BMG Labtech POLARstar Omega plater reader.

### Intraperitoneal Injection of Mice With Amyloid or Bacteria

NZBWF1/J (Jackson Labs, stock no: 100008) or Balb/cJ (Jackson Labs, stock no: 000651) mice, 6-8 weeks of age, were injected intraperitoneally with 50 µg of synthetic PSMα3 or PSMα3-DNA complexes twice weekly. Alternatively, mice were injected with 10^7^ *S. aureus* SH1000 or Δ*psm* mutant strains. Controls were injected intraperitoneally with 50 µL of sterile PBS. Blood was sampled weekly. Serum was collected by incubating blood for 30 minutes at 37°C and then centrifugation at 6000 rpm for 10 minutes. When applicable, 50 µl of blood was plated on tryptic soy agar to enumerate bacteria.

### Quantification of Bacteria on *S. aureus*-Colonized Mesh

Biofilms of *S. aureus* SH1000 and the Δ*psm* mutant grown on mesh in either tryptic soy broth or PNG were washed three times with sterile PBS to remove planktonic bacteria. Mesh were then placed in a sterile tube containing 3 ml PBS and sonicated for three intervals of 30 seconds at 30% amplitude. Prior experiments ensured that this sonication procedure did not kill the bacteria. Serial dilutions were used to enumerate bacteria released from the mesh.

### Mesh Insertion Into Mice

C57BL/6 wild-type or TLR-deficient mice were anesthetized with 2-4% isoflurane. The hair on the back flanks of the mice was removed. After sterilization of the site, a small incision was made using a scalpel. A 1 mm x 1 mm piece of mesh (sterile or colonized with biofilm) was inserted by creating a subcutaneous tunnel on the back flank. The incision was closed using a simple interrupted suture.

### Anti-dsDNA Autoantibody ELISA

The ELISA to quantify anti-dsDNA antibodies was performed as previously described (Gallo et al., 2015). Briefly, a 96-well plate (Costar, 07-200-33) was coated with 0.01% poly-L-lysine (Sigma, P8920) in PBS for 1 hour at room temperature. After coating, the plate was washed three times with distilled water. The plate was coated with 2.5 μg/mL calf thymus DNA (Invitrogen, 15633–019) in borate buffered saline (BBS; 17.5 g NaCl, 2.5 g H_3_BO_3_, 38.1 g sodium borate in 1 L H_2_O) and stored overnight at 4°C. The plate was washed three times with BBS and blocked with BBS containing 3% BSA and 1% Tween20 for 2 hours at room temperature. After washing five times with BBS, the plate was incubated with serial dilutions of control serum, naïve serum, or serum samples overnight at 4°C. After washing, biotinylated goat anti-mouse IgG (Jackson ImmunoResearch, 115-065-071) was added, and samples were incubated at room temperature for 2 hours with gentle rocking, and then incubated with avidin-alkaline phosphate conjugate (Sigma, A7294) at room temperature for 2 hours. Finally, the plate was washed five times with BBS and then incubated with 4-nitrophenyl phosphate disodium salt hexahydrate (Sigma-Aldrich, N2765) at a concentration of 1 mg/mL at room temperature protected from light. Optical densities were read using ELISA plate reader at 650 nm and 405 nm using a Molecular Devices Microplate Reader. Serum of a 6-to 8-week-old C57BL/6 mouse with no evidence of autoimmunity was used as a negative control for autoantibody production. As a positive control, serum from an MRL/lpr lupus-prone mouse, previously shown to have high levels of autoantibodies, diluted 1:250 in BBS, was used. All the samples shown in each figure were tested in the same ELISA assay, and the result are shown as raw optical density (O.D.).

### Statistical Analyses

Data were analyzed using GraphPad Prism software. One-way or two-way ANOVA with *post-hoc* Tukey multiple comparison tests were used as appropriate. The p values <0.05 were considered significant. *p < 0.05, ** p < 0.01, ***p < 0.001, and ****p < 0.0001 were marked in the figures.

## Data Availability Statement

The original contributions presented in the study are included in the article/**Supplementary Material**. Further inquiries can be directed to the corresponding author.

## Ethics Statement

The animal study was reviewed and approved by Institutional Animal Care and Use Committee at Temple University Lewis Katz School of Medicine.

## Author Contributions

All authors listed have made a substantial, direct, and intellectual contribution to the work and approved it for publication.

## Funding

CT is supported by NIH grants AI153325, AI151893, and AI148770. GW, JDA, EYL are supported by NSF DMR 1808459 and NIH R37 AI052453. JA is supported by NSF Graduate Research Fellowship Program DGE-1650604. EL acknowledges support from the Systems and Integrative Biology Training Program (NIH T32GM008185), the Medical Scientist Training Program (NIH T32GM008042) and the Dermatology Scientist Training Program (NIH T32AR071307) at the University of California, Los Angeles. EL also acknowledges an Early Career Research Grant from the National Psoriasis Foundation. Molecular graphics and analyses performed with UCSF Chimera, developed by the Resource for Biocomputing, Visualization, and Informatics at the University of California, San Francisco, with support from NIH P41-GM103311.

## Conflict of Interest

The authors declare that the research was conducted in the absence of any commercial or financial relationships that could be construed as a potential conflict of interest.

## Publisher’s Note

All claims expressed in this article are solely those of the authors and do not necessarily represent those of their affiliated organizations, or those of the publisher, the editors and the reviewers. Any product that may be evaluated in this article, or claim that may be made by its manufacturer, is not guaranteed or endorsed by the publisher.

## Supplementary Material

Supplementary Figure 1 Blood sampled from **(A)** NZBxW/F1 or **(B)** Balb/c mice implanted with *S. aureus* WT (black bars), Δ*psm* (blue bars) biofilm colonized mesh or control mesh (gray bars) was plated on tryptic soy agar and enumerated as colony forming units (CFU) per ml of blood. Mean and SEM graphed, two-way ANOVA using multiple comparisons used to determine significance. No statistical significance was determined.
