# Supplementary figures and images for: Phenol-Soluble Modulins From Staphylococcus aureus Biofilms Form Complexes With DNA to Drive Autoimmunity
Source: Front Cell Infect Microbiol. 2022 May 11;12:884065. doi: 10.3389/fcimb.2022.884065 (PMC9131096; doi:10.3389/fcimb.2022.884065)

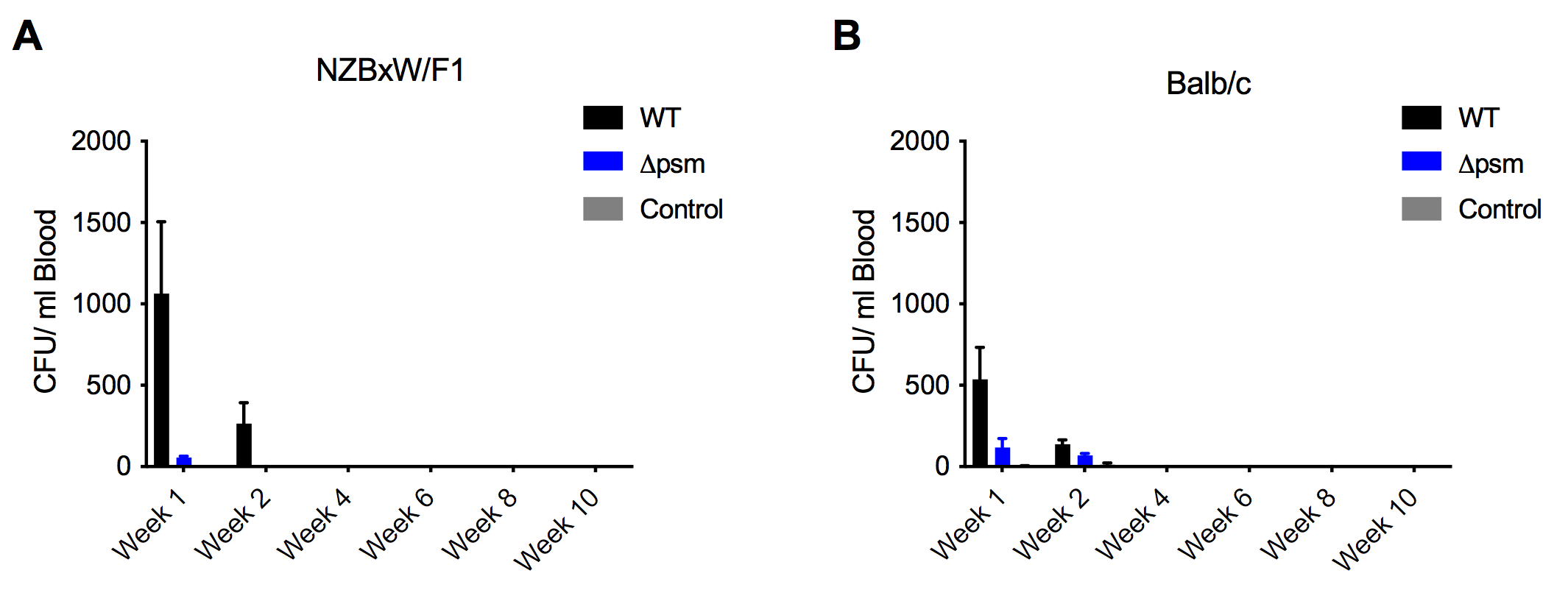

Supplement: Supplementary Figure 1 — Blood sampled from (A) NZBxW/F1 or (B) Balb/c mice implanted with S. aureus WT (black bars), Δpsm (blue bars) biofilm colonized mesh or control mesh (gray bars) was plated on tryptic soy agar and enumerated as colony forming units (CFU) per ml of blood. Mean and SEM graphed, two-way ANOVA using multiple comparisons used to determine significance. No statistical significance was determined. [file Image_1.tiff]
